# Strategies for improving physician documentation in the emergency department: a systematic review

**DOI:** 10.1186/s12873-018-0188-z

**Published:** 2018-10-25

**Authors:** Diane L. Lorenzetti, Hude Quan, Kelsey Lucyk, Ceara Cunningham, Deirdre Hennessy, Jason Jiang, Cynthia A. Beck

**Affiliations:** 1 Department of Community Health Sciences, Cumming School of Medicine, University of Calgary, 3330 Hospital Drive NW, Calgary, AB T2N4N1 Canada; 2 Department of Psychiatry, Cumming School of Medicine, University of Calgary, 3330 Hospital Drive NW, Calgary, AB T2N4N1 Canada

**Keywords:** Documentation, Emergency departments, Medical records, Physicians, Systematic reviews

## Abstract

**Background:**

Physician chart documentation can facilitate patient care decisions, reduce treatment errors, and inform health system planning and resource allocation activities. Although accurate and complete patient chart data supports quality and continuity of patient care, physician documentation often varies in terms of timeliness, legibility, clarity and completeness. While many educational and other approaches have been implemented in hospital settings, the extent to which these interventions can improve the quality of documentation in emergency departments (EDs) is unknown.

**Methods:**

We conducted a systematic review to assess the effectiveness of approaches to improve ED physician documentation. Peer reviewed electronic databases, grey literature sources, and reference lists of included studies were searched to March 2015. Studies were included if they reported on outcomes associated with interventions designed to enhance the quality of physician documentation.

**Results:**

Nineteen studies were identified that report on the effectiveness of interventions to improve physician documentation in EDs. Interventions included audit/feedback, dictation, education, facilitation, reminders, templates, and multi-interventions. While ten studies found that audit/feedback, dictation, pharmacist facilitation, reminders, templates, and multi-pronged approaches did improve the quality of physician documentation across multiple outcome measures, the remaining nine studies reported mixed results.

**Conclusions:**

Promising approaches to improving physician documentation in emergency department settings include audit/feedback, reminders, templates, and multi-pronged education interventions. Future research should focus on exploring the impact of implementing these interventions in EDs with and without emergency medical record systems (EMRs), and investigating the potential of emerging technologies, including EMR-based machine-learning, to promote improvements in the quality of ED documentation.

**Electronic supplementary material:**

The online version of this article (10.1186/s12873-018-0188-z) contains supplementary material, which is available to authorized users.

## Introduction

Chart accuracy is both a measure and a means of ensuring the quality of the care that patients receive [1]. Accurate patient chart information can facilitate and further communication between healthcare professionals involved in patient care, both in hospital and upon discharge into the community [2, 3]. Conversely, poor documentation can affect continuity of patient care, particularly during care transitions, and may cause delays or errors in patient treatment [4–8]. In 2018, a retrospective review of 138 antibiotic orders found that incomplete documentation resulted in longer median time to order resolution compared with completed documentation (31 vs 10 min, *p* = 0.02) [8]. During another retrospective chart review of 2061 patients who had undergone carotid enarterectomy, researchers found that charts deemed to be poorly documented were less freqently associated with appropriate scheduling of carotid endarterectomy procedures than charts of high quality (44.2% vs 52.9%, *p* < 0.001) [7]. As patient chart data is routinely used for hospital reimbursement, health system planning, resource allocation, and research activities, data quality may also impact outcomes beyond those associated with direct patient care [7, 9, 10].

Previous research suggests that considerable variation exists in the quality of physician documentation [7, 10, 11]. In the previously cited study of patients who underwent carotid endarterectomy, researchers, using a 10 point rating scale, found that of 2061 charts reviewed, only 42.6% were rated as well documented, with the percentage of high quality charts ranging from 14.6 to 87.5% across the 17 hospitals that were sampled [7]. Patient volume, care complexity, the variety and number of healthcare professionals involved in individual patient care, and the use of unformatted paper charts can all contribute to poor chart documentation [1, 6, 12].

Previous studies have demonstrated that a significant relationship exists between emergency department patient volume and errors or omissions in unformatted paper charts [13, 14]. Emergency Departments (ED) are characterized by frequent staff changes, high activity levels, overcrowding, frequent interruptions, time pressures, uncertain patient arrival patterns, and a wide variety of case presentations [14–17]. In such environments, fraught with risks for poor chart documentation, there is a pressing need for methods to better promote the recording of accurate and complete patient care information.

In recent years, electronic medical record (EMR) systems have been introduced into many EDs to facilitate the documentation of patient care episodes [18–21]. Despite this recent surge in EMR uptake, the quality of data in EMR systems remains variable [22, 23]. While some researchers have reported that EMRs improve guideline adherence, and reduce medication errors [18, 24], others claim that EMRs are but “clumsy electronic versions of paper charts” which, while increasing “the amount of information recorded”, do little to enhance the “readability” or overall quality of patient care information [25].

Research on the quality of EMR patient medical records tends to confirm that EMRs do not, by themselves, support enhanced physician documentation clarity, accuracy, completeness, or other measures of quality [26–31]. While some authors have found significant improvements in the accuracy, completeness, or richness (presence/completeness) of EMR documentation as compared with paper charts [26–29, 31], others have reported mixed results. In 2009, Alkasab et al. found that while EMR records of ED patient encounters included more clinical questions and information on medical histories, handwritten notes contained more data and details on non-significant improvements in symptoms and test results [26]. Perry et al. (2014) further noted that ED physicians spent significantly more time entering data into EMR applications as compared with paper charts [30].

Since EMR information is “necessarily documentation dependent”, strategies to enhance the quality of physician documentation can impact the accuracy, comprehensiveness, and usability of EMR records [32]. Further, as many emergency departments continue to rely on paper or hybrid charts, there is a broader need to identify and adopt effective approaches to documentation improvement that are not exclusively EMR-dependent. Such approaches may include: physician education, templates, dictation, and scanning of free-text paper notes into EMRs [33, 34].

The extent to which these and other interventions can improve documentation quality, particularly in the context of EDs, is, as yet, unclear. Thus, the objective of this study was to conduct a systematic review of the effectiveness of interventions to improve the quality of ED physician documentation in emergency settings.

## Methods

We searched the Cochrane Library, DARE Database of Reviews of Effects, EMBASE, MEDLINE, PubMED, and Web of Science to March 2015 to identify relevant English and French language peer reviewed literature suitable for inclusion in this review. No date limits were applied. Sources of grey literature, including the University of York’s Health Technology Database, Current Controlled Trials Register and the websites of government and professional organizations, were similarly searched. We also scanned the reference lists of included studies and review articles to identify additional studies of relevance to this review. This review was conducted in accordance with the Preferred Reporting Items for Systematic Reviews and Meta-Analyses (PRISMA) guidelines [35]. The protocol for this review has not been registered in PROSPERO or any other publicly accessible registry.

Search strategies combined search terms from two themes: 1) physicians (including but not limited to: clinician, physician, doctor, house officer, intern, resident, medical student) and 2) documentation (including but not limited to: administrative data, clinical coding, documentation, hospital record, medical chart). Terms were searched as both keywords and database subject headings as appropriate. No date limits were applied. An additional file is provided that outlines the search strategy used to identify relevant studies in the MEDLINE (OVID) database [see Additional file 1]. This MEDLINE (OVID) search was adapted to other electronic databases searched in this review. Copies of the complete search strategy are available, upon request, from the authors.

All abstracts were screened in duplicate, for inclusion in the full text review. Three authors independently screened the full texts of all selected abstracts. During both screening stages, disagreements were resolved through consensus. Studies were included if they reported on the results of any intervention to improve physician documentation in emergency settings. Studies were excluded if they 1) were descriptive studies or case reports, 2) reported only post-intervention results, 3) focused on populations other than physicians, residents or medical students, 4) centered on education to improve history taking, patient care for specific medical conditions, verbal communication skills, or the documentation of non-chart data, or 5) focused on documenting or evaluating student performance.

Four authors jointly extracted data from all included studies into a standardized form created in Excel. Outcomes of interest included: documentation accuracy, clarity (understandability), legibility, completeness, presence, and timeliness. Two authors independently assessed the quality of included studies, using the Downs and Black checklist of 27 quality criteria for randomized and non-randomized designs [36]. Due to the heterogeneity of study designs, and outcomes, it was not possible to pool the data from included studies.

## Results

**Fig. 1:**
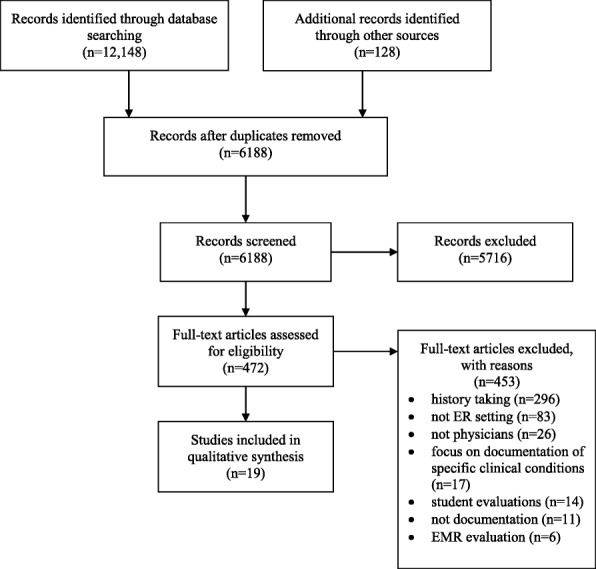
PRISMA Flow Diagram

A total of 6188 unique abstracts were identified from electronic database and other searches. Four hundred and seventy-two of these were selected for full text review, 19 of which were deemed appropriate for inclusion in the final review (Fig. 1). One RCT, 6 quasi-experimental, and 12 pre-experimental (cross-sectional, or pre-post with no comparison) studies evaluated interventions to improve physician documentation in ED teaching and non-teaching hospitals and trauma centers in Australia (*n* = 3), Belgium (*n* = 1), Canada (*n* = 3) New Zealand (*n* = 1), United Kingdom (*n* = 3) and the United States (*n* = 8) (Table 1).

**Table 1:** Characteristics and Outcomes of Included Studies

Author Date Country	Study design^a^	Setting Participants	Intervention(s) Duration	Intervention & Control Group Details	Outcomes of Interest	Results	Downs & Black Quality Score
Carter et al. USA (2009)	PPN	• Teaching hospital • Residents (R2, R3)	Multiple Intervention (audit/feedback, education, and reminders) 12 weeks	Intervention group *n* = 24 1-h lecture to 18/24 residents Pocket card and lecture handouts to 24/24 residents and biweekly newsletters. Physicians received weekly case specific chart audit/feedback. Control group (n = 24) Usual electronic documentation program	• Chart level, based on complexity of decision making and detail of history and physical. • RVU (relative value units). • Billings/hr.	• Intervention resulted in more complex charting (27% vs 19%, *p* < .01) and fewer mid-level charts (*p* < .01). • RVUs increased with intervention (3.71 vs 3.17, p < .01). • Billings increased with intervention ($354.08 vs $303.79, p < .01).	19/27
Cole & Counselman USA (1995)	RFUP	• Teaching hospital • Residents & Physicians	Dictation 50 weeks	Intervention group *n* = 94 Dictated report Dictation services available for 8 h per day, alternating between day and evening shifts. Control group *n* = 108 Usual paper charts	Mean number of 28 critical items present in report.	Significant mean increase in the number of critical items reported (19.6 vs 15.8; p < .01).	19/27
De Winter et al. Belgium (2011)	PPN	• Teaching hospital • General internist and internal medicine trainees	Reminder 14 weeks	Intervention group *n* = 924 A limited questions list to encourage collection of data on patients’ prescription and non-prescription medications. Pharmacists interviewed patients to collect complete medication histories for gold standard comparison. Control group *n* = 798 Usual paper chart	Proportion of drugs omissions in physician history compared to pharmacy-technician gold standard history taking.	Significant decrease in the proportion of drug omissions (9% vs. 17%, *p* < .001).	20/27
Dexter et al. UK (2008)	PFUP	• Otolaryngology Emergency Clinic • “Doctors”	Multiple Intervention (education and template) Not specified	Intervention group n = 140 Proformas to encourage documentation of patient information. Advice provided on how to improve handwriting. Case notes audited using an ANKLe (Adjusted Note Keeping and Legibility) scoring system. Control group *n* = 140 Usual documentation	Legibility, content, and ANKLe (Adjusted Note Keeping and Legibility) scores.	Significant improvements in mean ANKLe scores for note content (17.2 vs. 16.0, *p* < 0.05), legibility (3.02 vs. 2.96, *p* < 0.05) and overall ANKLe score (20.24 vs. 18.95, p < 0.05).	15/27
Goodyear et al. UK (1995)	CSC	• Emergency Department • “Junior doctors”	Template 30 weeks	Intervention group *n* = 100 Pre-printed pediatric admission assessment forms. Control group *n* = 100 Usual handwritten medical records.	Mean numbers of 25 core clinical details recorded: mean number of words per clerking.	Significant increase in number of core clinical details recorded - 24 recorded with intervention vs. 17.6 (*p* < 0.001) and words per clerking 144 for intervention vs. 184 (*p* < 0.001).	12/27
Hanson et al. UK (1994)	TSS	• 2 Teaching hospitals • House Officers	Audit/Feedback 19 weeks	Intervention group *n* = 420(Feedback 1); 429(Feedback 2); 244 (Final audit – weeks 20–24) Phase I: Feedback at 6 weeks in form of individual audit/feedback and group discussion. Phase II: Feedback at week 11. Further audit during weeks 11–16. Post-intervention final audit weeks 20–24. Control group *n* = 401 No feedback. Baseline audits. Usual paper charts.	• Proportion of head injury charts documenting GCS (Glascow Coma Scale). • Proportion of charts documenting diagnostic coding for all patients.	• Significant improvement in GCS documentation for both hospitals for patients with head injuries during all phases of the study – (80% Feedback 1, 88% Feedback 2, 90% Final Audit vs 40% at baseline). • Significant improvement in diagnostic coding for Hospital A from baseline (*p* < .008).	18/27
Heidt & Griffey USA (2012)	PPN	• Teaching hospital • Emergency Physicians	Audit/Feedback 12 weeks	Intervention group *n* = 382 Individualized email feedback from coders to physicians whose charts lacked sufficient documentation to warrant the inclusion of critical care billing codes Control group *n* = 501 No feedback.	Proportion of ICU (intensive care unit) admissions that documented critical care time.	Significant increase in the number of charts documenting critical care time (64% vs 18%, p < .001).	10/27
Humphreys et al. USA (1992)	CSC	• Teaching hospital • Internal medicine housestaff and ED physicians	Template 31 weeks	Intervention group *n* = 99 Preformatted chart for obstetric or gynaecological problems. Control group *n* = 60 Standard blank charts.	Proportion of ICU (intensive care unit) admissions that documented critical care time in the emergency room.	Significant increase in documentation of critical care time (243/382 (64%) vs 88/501 (18%) - p < 0.001).	22/27
Kondziolka et al. Canada (1989)	PPN	• Regional trauma unit • Physicians	Template Not specified	Intervention group n = 100 Neurotrauma Assessment Record templates with 32 information parameters. Control group n = 100 Usual paper chart	Proportion of charts with each of 32 assessed items.	Significant improvement in the recording of elements including incident time and transfer, and medical history (p < .001), and a significant decrease in recording of treatment plans (*p* < .001).	19/27
Marill et al. USA (1999)	RCT	• ER trauma centre • Physicians, Residents & Medical Students	Template Approx. 2.5 weeks (16 days)	Intervention group *n* = 657 Commercial templates-guided medical documentation system for all patients presenting to ERs during a 16 day period. Control group *n* = 570 Usual paper chart	• Emergency physician total treatment and evaluation time. • Total professional bill and physician satisfaction with documentation method.	• Non-significant reduction of 4.6 min in treatment time (95% confidence interval [CI], −9.2 to 18.3). • Significant mean increase in total billing ($137.40 vs $107.80; 95% CI for difference - $22.20 to $37.00).	25/27
O’Connor et al. New Zealand (2001)	PPN	• Non-teaching rural hospital • Physicians	Template 2 weeks	Intervention group *n* = 96 Preformatted emergency department charts with 8 key content items Control group *n* = 137 Usual paper charts	• Median number of parameters filled in for each chart, out of 8. • Proportion of charts recording each of 8 parameters.	• Significant mean increase in the number of parameters documented in each chart (8 vs 7, *p* = .005). • Significant positive change in the recording of one parameter – Physician Name (52% vs 18%, *p* < .0001).	19/27
Otillo et al. USA (2014)	PPC	• Academic children’s hospital • Pediatric residents	Education 112 weeks	Intervention group *n* = 157 One-hour lecture Control group *n* = 145 No education	Proportion of charts with documentation of 3 specific findings.	No change in right lower quadrant tenderness documentation (for example): 43.9% vs. 35.9%, 95% CI -19 to + 3	20/27
Schnieden & Good Australia (1996)	PPN	• Emergency department • House Officers & Physicians	Template 20 weeks	Intervention group n = 50 Psychiatric assessment templates Control group *n* = 50 Usual paper charts	Median score (max = 100) for adequacy of documentation of 25 items in history, exam, and treatment).	• Significant increase in median score (33 vs 18; p < .01). • Significant increase in proportion of charts documenting education (*p* = .029), alcohol (*p* = .045), smoking (*p* = .009) and interview alone (*p* = .0001). Non-significant changes for remaining topics. • Overall increase in psychosocial history documentation (9% vs > 1%, *p* = .003) • Overall increase in newly documented psychosocial problems (16% vs. 10%, *p* = .05).	18/27
Teo et al. Australia (1995)	PFUP	• Paediatric emergency department • Physicians	Multiple Intervention (education, reminder, and template) 5 weeks	Intervention group *n* = 52 Phase I: Education and reminders to increase the quality of pediatric asthma documentation (2 weeks). Phase II: Physicians mandated to adopt an acute asthma proforma (3 weeks) Control group *n* = 204 Usual paper charts. No specific education.	Proportion of charts documenting each of 19 items	• Phase I intervention – education and reminders – resulted in no statistically significant change in documentation. • Phase II intervention – Template/Proforma – significantly improved documentation of 8 of 19 items (p < = .03).	17/27
Van Amstel et al. Canada (2004)	PPN	• Pediatric teaching hospital • Physicians	Reminder/ 4 weeks	Intervention group n = 153 HEADSS (Home, Education, Alcohol, Drugs, Smoking, Sex) stamp in patient charts to remind physicians to document these data items in charts. Control group *n* = 153 Usual paper charts	• Difference in proportion of charts containing information on psychosocial problems related to: Home, Education, Alcohol, Drugs, Smoking, Sex • Extent of global documentation • Proportion of charts with newly documented psychosocial problems in the above focus areas.	• Significant increase in proportion of charts documenting education (p = .029), alcohol (p = .045), smoking (p = .009) and interview alone (p = .0001). Non-significant changes for remaining topics. • Overall increase in psychosocial history documentation (9% vs > 1%, p = .003) • Overall increase in newly documented psychosocial problems (16% vs. 10%, p = .05).	20/27
Vasileff et al. Australia (2009)	PPN	• Teaching hospital • Emergency department doctors	Facilitation (pharmacist medication verification) 5 weeks	Intervention group *n* = 29 Pre-admission medication history documented on patients’ charts by pharmacists (and verified by patient’s pharmacy) before patients were seen by emergency department physicians. Control group *n* = 45 Physicians documented pre-admission medications on a standard form.	• Discrepancies in documented medication histories • Medication errors	• Overall decrease in unintentional medication discrepancy in patients: 3.3% vs. 78.6% (p < 0.05) • Decrease in average number of discrepancies per patient 0.03% vs. 2.51% (*p* < 0.05). Reduction of missed doses of pre-admission medications 0 vs. 1.04 (p < 0.05)	13/27
Voaklander et al. Canada (2000)	PPC	• Teaching hospital • Emergency department physicians	Multiple Intervention (education and reminder) 13 weeks	Intervention group *n* = 321 flagged charts; 323 un-flagged charts Injury surveillance training, pocket reminder cards, modification of existing emergency department charts to include chart reminder labels, and space added for inclusion of additional injury related data Control group *n* = 645 Handwritten unmodified charts	Presence of 14 key data elements included in education intervention	• Significant increase in mean number of 10 of 14 documented data elements - 8.1 flagged charts vs 7.3 unflagged charts vs 6.9 pre-intervention (p < 0.05). • Significant decrease in documentation of prevention measures: (12.1% vs. 21.4%) OR 0.56 (0.38–0.83 CI *p* > 0.05). • Significant increase (post-intervention) in documentation of activity at time of injury, location of injury, address where injury occurred, adult observer present and environmental conditions (p < 0.05)	19/27
Wrenn et al. USA (1993)	PPN	• Teaching hospital • House staff	Template 35 weeks	Intervention group *n* = 1129 Structured complaint-specific patient encounter forms for laceration, closed-head injury, pharyngitis, and asthma available to all ER house-staff for 8 months. Control group *n* = 1276 Usual paper charts	• Proportion of charts documenting 30 aspects of history, physical and treatment • Proportion of charts with complete prescription information	• Mixed results, reported as percentages and odds ratios, across 30 parameters of history taking, physical and treatment – range: 97% vs 17% (OR 176, p < .001) to 98.4% vs 94.4% (OR .28 p: NS) • Significant increase in proportion of charts documenting prescription information (80% vs 73%, *p* = .007)	19/27
Zick & Olsen USA (2001)	PPN	• Suburban level 1 trauma centre • Physicians	Dictation Not specified	Intervention group n = 47 Dragon Naturally Speaking voice recognition software. 30 min training for physician. Control group *n* = 47 Traditional voice transcription services	Difference in accuracy (per cent of words correct in document).	Decrease in accuracy of words documented (98.5% vs 99.7% - change of −1.2; CI (−1.5 to −0.8))	17/27

^a^*CSN* cross sectional study with control, *PFUP* prospective follow-up with comparison, *PPC* pre-post comparison, *PPN* pre-post no comparison, *RCT* randomized controlled trial, *RFUP* retrospective follow-up with comparison, *TSS* time series study

The quality of included studies, per the Downs and Black scale, ranged from low < 15 (*n* = 3), to moderate 15–19 (*n* = 11), to high > 19 (*n* = 5) (Table 1). Quality issues identified in most studies included the absence of descriptions of principle confounders (*n* = 10), and lack blinding of participants (*n* = 14) or those assessing the outcomes of interventions (*n* = 17).

Seven interventions were identified to improve physician documentation in ED settings. These included: audit/feedback (*n* = 2), dictation (*n* = 2), education (*n* = 1), facilitation (*n* = 1), reminders (*n* = 2), structured paper templates (*n* = 7), and multi-interventions (*n* = 4) that incorporated two or more approaches to improving documentation (Table 1).

### Audit/feedback

Two studies (one time series, one pre-post without control) explored the impact of audit/feedback on improving ED documentation [37, 38]. In both studies, audit/feedback significantly improved the richness (presence and completeness) of physician documentation (Table 1).

### Dictation

Two studies (one pre-post without control; one retrospective follow up with control) investigated dictation as a means of improving documentation quality [39, 40]. (Table 1) In a retrospective comparison of dictated and paper charts, Cole and Counselman (1995) reported significant improvements in the number of 28 critical items documented for patients presenting with chest pain [39]. Zick and Olsen (2001) compared voice recognition software (Dragon Naturally Speaking®) to traditional dictation. While completion time decreased with the use of voice recognition software (Dragon Naturally Speaking®) so too did the overall accuracy of physician documentation [40].

### Education

One study (pre-post with control) evaluated the effectiveness of an education intervention, a 1 h lecture on medical liability, to improve residents’ documentation of pediatric emergency charts [41]. Researchers found no difference in the richness (presence and completeness) of the charts documented post-lecture (Table 1).

### Facilitation

One study (pre-post without control) evaluated the effects of a pharmacist intervention on the quality of physician documentation [42]. Researchers determined that pharmacists’ involvement in recording medication histories for older patients taking 4 or more concurrent medications resulted in significantly fewer unintentional discrepancies in patients’ usual drug regimens, and missed or incorrect medication doses prescribed to patients [42].

### Reminders

In two studies (both pre-post without controls), researchers found that reminders, in the form of physician question lists or chart stamps, significantly improved the quality (presence of specified items) of physician documentation when compared with unformatted paper charts [43, 44]. While De Winter et al. (2011) reported a significant decrease in the number of drug history omissions, Van Armstel et al. (2004) noted a significant increase in overall documentation [43, 44].

### Templates/forms

Seven studies (two cross-sectional control, four pre-post without control, one RCT) compared templates to unformatted paper charts [25, 45–50]. Three studies (two cross-sectional control, one pre-post without control) reported significant improvements in physician documentation [45, 46, 49]. The remaining four studies (three pre-post without control, one RCT) reported mixed results in intervention effectiveness [47, 48, 50, 51]. Kondziolka et al. (1989) noted improvements in recordings of incident time, patient transfer time, and medical history, but a decrease in the presence of patient treatment plans [47]. O’Connor et al. (2001) found that, while templates resulted in a significant mean increase in a number of “key content items,” the only consistent improvement was in the recording of physicians’ names [48]. Wrenn et al. (1993) reported that templates significantly improved physicians’ recording of patient prescription information, yet achieved mixed results in the documentation of 30 items relevant to history taking, physicals, and the treatment of patients with asthma, lacerations, pharyngitis, or closed head injuries [50]. Finally, a randomized controlled trial by Marill et al. (1999) found that the use of templates did not significantly reduce patients’ time to treatment [51].

### Multiple interventions

Four studies (one pre-post without control, one pre-post control, two prospective follow up with comparison) assessed the impact of multi-interventions on the quality of physician documentation [52–55]. While all studies included an education component, they varied in the type and number of other interventions that were included. One study included audit/feedback and reminders; one templates; one reminders; and, one reminders and templates. Two of the four studies (one pre-post without comparison, one prospective follow up) reported positive results associated with the use of multi-interventions to improve documentation quality. Carter et al. (2009) found improvements in the completeness of physicians’ chart documentation as a result of lectures, pocket reminders, and case-specific chart audit/feedback, and Dexter et al. (2008) reported that templates and education enhanced the richness (presence and completeness) and legibility of physicians’ chart documentation in an otolaryngology emergency clinic [52, 53]. In contrast, Teo et al. (1995) found that, while the introduction of templates did improve physician documentation of required items for asthma diagnosis and reporting in a pediatric emergency department, education and reminders did not result in any significant increase in the presence and completeness of asthma documentation [54]. Finally, Voaklander et al. (2000) reported that pocket cards and chart labels significantly increased the number of items recorded for pediatric injuries, including activity at the time of injury, yet decreased documentation of injury prevention measures [55].

## Discussion

Accurate and complete physician documentation is essential to ensuring that patients receive appropriate and timely care [56]. In environments where increasing numbers of healthcare organizations are digitizing patient health data and enabling data sharing among healthcare providers and health researchers, it is increasingly essential to ensure that these data are of the highest quality. Our study identified a number of promising strategies to improve the accuracy, completeness, and overall quality of physician documentation in EDs with and without access to EMRs. These included audit/feedback, pharmacist-led medication reconciliation, paper or electronic templates, and multi-pronged education interventions. To our knowledge, this is the first systematic review of the effectiveness of interventions to improve ED documentation.

Our findings mirror those of related studies on the effectiveness of interventions to improve written and verbal communication in hospital settings [3, 5, 57]. Prior research in non-ED settings indicates that active (e.g. audit/feedback or templates) and/or multifaceted interventions that explicitly engage participants may be more effective than passive interventions (e.g. printed education materials) in effecting lasting changes in physicians’ documentation behavior [15, 58–61]. While the findings from our review tend to confirm that active interventions such as audit/feedback, reminders, or templates and/or multiple interventions may improve physician documentation, we did observe mixed results with respect to documentation comprehensiveness and accuracy when these interventions were introduced into ED settings [26, 44, 47, 48, 50, 51, 54, 55]. The quality of the studies identified in this review, and variability in settings, outcome measures, and intervention duration across studies may have contributed to this finding; when appropriately designed for the context or environment in which they will operate, these interventions may prove even more effective in supporting improvements in physician documentation.

While many emergency settings are currently using, or considering implementing, electronic medical documentation systems to track patient care, the accessibility, usability, and time required to use EMR systems can, particularly in high pressure environments such as EDs, present barriers to achieving improvements in both physician documentation and associated patient care [34]. Although EMRs and other technologies may facilitate improvements in the quality of ED physician documentation, it is ultimately how these technologies are designed, implemented, and used that will determine their effectiveness [62]. For example, while many EMRs systems incorporate electronic templates into their design, templates do not, as this review suggests, in and of themselves guarantee the comprehensiveness and accuracy of the documentation recorded therein. Similarly, the mere presence of an EMR system does not automatically improve the quality of ED patient data; indeed, EMRs can perpetuate, even exacerbate, existing deficits in physician documentation [34]. When inadequately designed, implemented, or used, they may be no better, and perhaps worse, than unformatted paper charts [22, 23, 34, 63–65]. Further, as many emergency physicians continue to rely on paper charts or hybrid systems to record, track, and communicate the progress of patient care, no one documentation-improvement strategy may be effective in all settings.

Successful approaches will likely be those that can adapt to different settings, be seamlessly integrated into existing workflows, and garner widespread acceptance from all relevant stakeholders [66]. While our review found that interventions such as structured templates can improve the quality of paper and presumably electronic documentation, the increasing adoption of EMRs in EDs and other healthcare settings continues to require and inspire the development of a multitude of innovative solutions to facilitate the timely creation of comprehensive and accurate patient records [67]. For example, medical scribes, or “nonlicensed health care team members that document patient history and physical examination contemporaneously with the encounter” have been incorporated into EDs and other settings to improve the speed and comprehensiveness of physician documentation [68]. While studies evaluating the impact of medical scribes have reported positive outcomes with respect to patient flow and patient-provider satisfaction outcomes, medical scribes may also promote the creation of comprehensive high-quality patient care records [68, 69]. Traditional health information management specialists can also play a role in monitoring and promoting the quality of both paper and EMR records.

Opportunities also exist to incorporate advances in artificial intelligence and machine learning into chart documentation processes [70, 71]. In the future, it may be possible to embed artificial intelligence technologies, including machine learning, into EMR systems to alert physicians to patient information or physician orders that are potentially inaccurate, imprecise, incomplete, or inappropriate [72–79]. In Alberta, Canada efforts are underway to implement an EMR that will allow health information management specialists to conduct automatic documentation checks prior to patient visits, during care, and post discharge [80]. These checks will facilitate the consolidation of medication lists and allergies, and identify and alert physicians in real time to missing and incomplete chart information, and/or contradictions between patient histories and orders for medications or investigations.

While our review found little evidence of the effectiveness of speech recognition software in ED settings, recent advances in software development, machine learning, and bedside data capture suggest that effective, reliable, and efficient approaches to improving the quality of electronic patient notes may soon become commonplace [81–83]. In a recent study, Payne and colleagues described the development of a mobile app to convert voice-recorded patient notes into EMR-compatible text [83]. Other studies have reported on the development of automated or “digital scribes” to record and convert speech to text [81, 82]. Researchers at Stanford University (United States) are designing “digital scribe” software that will incorporate artificial intelligence and voice recognition to enable physicians to create comprehensive, quality patient data that can be uploaded to EMR systems in real time [81]. Other researchers at the University of California at Berkley (United States) have also described the creation of a prototype “automated medical scribe” that relies on “speech-processing modules” to “convert a transcribed spontaneous conversation into a concise and fully formatted report” [82]. If successful, these and similar initiatives may facilitate the creation of quality of electronic patient data, while simultaneously reducing administrative and workflow burdens associated with EMR systems [84].

Although ongoing technological advances may radically improve the quality of physician documentation, it is important to note that transforming documentation practices also requires changing learned behavior, or habits. Habits are patterns of behavior that are “acquired through incremental strengthening [repetition] of the association between a situation (cue) and an action [85].” As habits are formed over sustained periods of time, interventions designed to alter behavioral norms should similarly be of long duration. In a recent study on habit formation, researchers determined that while some individuals can easily adopt new personal and professional habits, others require much longer periods of time to alter behavioral norms [85]. Behavioral theorists further suggest that personal and environmental factors, including individual motivation and organizational culture and norms, can profoundly impact behavior change [86, 87]. Thus, the extent to which improvements in ED documentation are valued above other competing personal or organizational objectives, such as organizational expectations with respect to patient turnover, may affect the uptake and impact of these interventions. To effect lasting and meaningful improvements in ED documentation, it may be necessary to directly involve all stakeholders, including physicians and residents, in selecting, contextualizing, implementing, and conducting ongoing evaluations of multifaceted approaches to improving documentation quality in EDs [57, 61].

This study has caveats and limitations. While we employed an extremely comprehensive search strategy, inconsistencies in the indexing of studies in electronic databases and our decision to restrict our search to English or French language publications, may have impacted on our ability to identify all relevant studies. Further, variability in study design and outcomes assessed across studies limited our ability to quantitatively compare the outcomes from individual studies, and assess the overall effectiveness of many of these approaches. Finally, the preponderance of pre-experimental studies (*n* = 18) included in this review suggests that the literature on ED physician documentation improvement is yet in its infancy, and that further research is required to determine how best to encourage documentation improvements in these settings.

## Conclusions

As more hospitals and primary care centers implement EMRs, healthcare providers, researchers, and decision makers will increasingly rely on patient health records to facilitate patient care, research, and health system planning; the need for accurate and complete physician documentation will only increase. At the systems level, documentation quality should and can be an indicator of health system performance. Achieving improvements in physician documentation is a complex process, requiring the active support of various stakeholders, and the implementation of systems that can be adapted to the demands of existing workflows, and the availability of adequate ongoing training [88]. While this review suggest that audit/feedback, reminders, templates and/or multiple interventions are potentially promising approaches to improving physician documentation, further research is needed to confirm these findings, and explore other approaches, including machine-learning and other emerging technologies, to advance ongoing improvements in physician documentation in ED settings.

## Additional file

Additional file 1: MEDLINE Search Strategy. Copy of the search strategy used to identify relevant studies in the MEDLINE (OVID) database. (DOC 25 kb)

## Abbreviations

CSN: Cross sectional study with control
ED: Emergency Department
EMR: Electronic medical record
PFUP: Prospective follow-up with comparison
PPC: Pre-post comparison
PPN: Pre-post no comparison
PRISMA: Preferred Reporting Items for Systematic Reviews and Meta-Analyses (PRISMA)
RCT: Randomized controlled trial
RFUP: Retrospective follow-up with comparison
TSS: Time series study
