# Supplementary material for: Strategies for improving physician documentation in the emergency department: a systematic review
Source: BMC Emerg Med. 2018 Oct 25;18:36. doi: 10.1186/s12873-018-0188-z (PMC6297955; doi:10.1186/s12873-018-0188-z)
Supplement: Supplementary file 1 — MEDLINE Search Strategy. Copy of the search strategy used to identify relevant studies in the MEDLINE (OVID) database. (DOC 25 kb) [file 12873_2018_188_MOESM1_ESM.doc]

**MEDLINE (OVID 1946 to March 29 2015) Search Strategy**

1. exp Medical Records/cl, mt, og, st [Classification, Methods, Organization & Administration, Standards]

2. *medical history taking/

3. *Diagnosis-Related Groups/cl, mt, og, ut [Classification, Methods, Organization & Administration, Utilization]

4. ((EMR or EHR) adj3 data).tw.

5. 1 or 2 or 3 or 4

6. exp physicians/ or students, medical/ or "internship and residency"/

7. (physician* or doctors or house staff or housestaff or house officer* or intern or interns* or medical officer* or medical staff or clinical staff or medical resident* or medical residency or medical student*).tw.

8. (fellow or fellows or house staff or housestaff or house officer* or medical officer*).tw.

9. 6 or 7 or 8

10. 5 and 9

11. clinical coding/

12. ((physician* adj3 documentation) or clinical coding or discharge summaries).tw.

13. ((physician* or doctors or house staff or housestaff or house officer* or intern or interns* or medical officer* or medical staff or clinical staff or residents or medical residency or medical student*) adj5 ((administrative adj3 data*) or clinical coding or history taking or medication record* or observation chart* or operative report* or hospital data* or hospital record* or medicaid claim* or medical record* or discharge data or discharge record* or discharge summar*)).tw.

14. ((physician* or doctors or house staff or housestaff or house officer* or intern or interns* or medical officer* or medical staff or clinical staff or residents or medical residency or medical student*) adj5 ((EMR or EHR or medical or clinical or hospital) adj3 (charting or coding or document* or note entr* or orders))).tw.

15. 10 or 11 or 12 or 13 or 14

16. limit 15 to (english or french)
